# Facebook Groups as a Powerful and Dynamic Tool in Medical Education: Mixed-Method Study

**DOI:** 10.2196/jmir.7990

**Published:** 2017-12-22

**Authors:** Leo Nicolai, Moritz Schmidbauer, Maximilian Gradel, Sabine Ferch, Sofía Antón, Boj Hoppe, Tanja Pander, Philip von der Borch, Severin Pinilla, Martin Fischer, Konstantinos Dimitriadis

**Affiliations:** ^1^ Institute for Medical Education University Hospital Ludwig-Maximilians-University Munich Munich Germany; ^2^ Department of Neurology University Hospital Ludwig-Maximilians-University Munich Munich Germany

**Keywords:** social media, Facebook, medical education, e-learning, faculty, networking

## Abstract

**Background:**

Social networking sites, in particular Facebook, are not only predominant in students’ social life but are to varying degrees interwoven with the medical curriculum. Particularly, Facebook groups have been identified for their potential in higher education. However, there is a paucity of data on user types, content, and dynamics of study-related Facebook groups.

**Objective:**

The aim of this study was to identify the role of study-related Facebook group use, characterize medical students that use or avoid using Facebook groups (demographics, participation pattern, and motivation), and analyze student posting behavior, covered topics, dynamics, and limitations in Facebook groups with regards to educational usage.

**Methods:**

Using a multi-method approach (interviews, focus groups, and qualitative and quantitative analysis of Facebook posts), we analyzed two representative Facebook groups of medical preclinical semesters at Ludwig-Maximilians-University (LMU) Munich. Facebook primary posts and replies over one semester were extracted and evaluated by using thematic content analysis. We developed and applied a coding scheme for studying the frequency and distribution of these posts. Additionally, we interviewed students with various degrees of involvement in the groups, as well as “new minorities,” students not registered on Facebook.

**Results:**

Facebook groups seem to have evolved as the main tool for medical students at LMU to complement the curriculum and to discuss study-related content. These Facebook groups are self-organizing and quickly adapt to organizational or subject-related challenges posed by the curriculum. A wide range of topics is covered, with a dominance of organization-related posts (58.35% [6916/11,853] of overall posts). By measuring reply rates and comments per category, we were able to identify learning tips and strategies, material sharing, and course content discussions as the most relevant categories. Rates of adequate replies in these categories ranged between 78% (11/14) and 100% (13/13), and the number of comments per post ranged from 8.4 to 13.7 compared with the average overall reply rate of 68.69% (1167/1699) and 3.9 comments per post. User typology revealed social media drivers (>30 posts per semester) as engines of group function, frequent users (11-30 posts), and a majority of average users acting rather as consumers or lurkers (1-10 posts).

**Conclusions:**

For the moment, the medical faculty has no active involvement in these groups and therefore no influence on accuracy of information, professionalism, and ethical issues. Nevertheless, faculty could in the future benefit by extracting relevant information, identifying common problems, and understanding semester-related dynamics.

## Introduction

### Use of Social Networking Sites

Social networking sites (SNSs), led by Facebook with almost two billion monthly active users worldwide in early 2017, transform the way we perceive the world, as well as how we communicate and socialize [1,2]. In the field of medicine, the effect of SNSs can be particularly far-reaching, for example, by changing the doctor-patient relationship [3,4]. Given the fact that a high percentage of university students actively use Facebook and related services, SNSs have also started to change (medical) student and university life [5,6].

Different people use Facebook for different purposes. Kumar et al (2010) described three roles people can take in regards to social media evolution: (1) *passive members (lurkers)*, (2) *inviters,* and (3) *linkers* [7].

According to Stutzman et al (2006), individuals use Facebook for leisure, to learn about each other, or for communication purposes [8]. Mazmans and Usluels (2010) structural model for Facebook usage describes four main purposes: (1) social relations, (2) work-related, (3) daily activity, and (4) educational usage [9]. Facebook users can exchange information through chat, post information on their personal profiles or profiles of others, look at profiles of peers, share multimedia content, or organize themselves in public, closed, or secret groups.

### Social Networking Sites in Education

These technical features make effective aggregation and modification of knowledge and information possible; they make connectivity and social support easier and therefore, contribute significantly to the creation of new content. For these exact reasons, Lee and McLoughlin (2008) also identified social media as pedagogical tools [10]. Collaboration, communication, material sharing, peer feedback, and social media prowess are considered the main factors important for educational usage of a social media platform [9,11-13].

Some researches compare social media platforms, such as Facebook, to web based educational tools [12]. Others have recognized SNSs as an instrument to teach and educate, focusing on e-learning and interaction with faculty [9,11]. Pilot studies have already utilized Facebook for educational purposes at medical schools, for example, to complement university courses [14,15]. Selwyn (2009) analyzed activities on students’ Facebook walls of over 900 undergraduate students to identify different types for educational activity and defined four main categories: (1) recounting and reflecting on university activity, (2) exchange of practical information, (3) exchange of academic information, and (4) displays of supplication or disengagement [9,11].

### The Role of Facebook Groups

However, the educational use of SNSs and its effects are still insufficiently examined. Although Facebook groups have been identified as being useful in faculty-rooted course support, as well as representing organic (self-organizing) student-based learning environments [16], and although these groups combine many of the aforementioned attributes essential for educational usage, very little is known about which students use these groups, how and for what exact purpose [17]. Some researches argue that certain learning styles are more beneficial for learning through SNSs, but further research is necessary to examine these findings in different settings [18-22].

Facebook groups allow a quick and easy organization of individuals with related interests or characteristics, who can then share posts, pictures, and material on the group’s wall [23].

Their technical features have led to some excitement among educators, as they provide a student-centered platform ideally suited for peer-generated content, peer-to-peer communication, and learning and interactive support, combined with social aspects such as peer-mentoring and personal interaction and bonding [24,25].

### Possible Limitations of Social Networking Sites in Education

However, SNS use in education comes with relevant caveats, as some studies hint at problems in structure and (self-) organization, domination of groups and discussions by individuals, feelings of incapability by weaker users, and deviation from educational goals [26,27]. Scripted cooperation to better structure discussions as suggested by computer-supported, cooperative learning–related research did not seem to be very efficient in an SNS context so far.

Measurement of benefits has been elusive, and various pitfalls and dangers of SNS integration into curricula have been reported, for example, privacy issues, online misconduct, and the so-called *digital divide*, excluding individuals who do not have access to SNSs [28-30]. Moreover, one study found Facebook to be a place for reflection on and criticism of study-related content by undergraduate students, joining in with other studies which report students to be wary and opposed to faculty involvement in SNSs [31].

### Aim of This Study

In light of these findings, however, a clear picture on educational usage of Facebook groups has yet to emerge to assess to what extent medical faculties can take educational advantage of these networks. In particular, there is a lack of data on user motivation, user typologies, subjective benefits, and limitations, as well as patterns of posting behavior of students necessary for further analysis and integration into existing (learning) theories [32]. We therefore designed this explorative study to identify the role of study-related Facebook group use; characterize medical students that use or avoid using Facebook groups (demographics, participation pattern, and motivation); analyze covered topics quantitatively and qualitatively; describe dynamics within the groups, as well as posting patterns; and define limitations of Facebook groups with regards to educational usage.

## Methods

### Research Setting

The medical faculty at LMU Munich offers a unique opportunity of further research in the field of educational usage of social media, as the majority of medical students of each preclinical year join year and cohort specific, semester-spanning Facebook groups (ie, named “LMU medical students starting in winter semester 12/13,” used throughout the medical studies of the respective cohort).

We identified two relevant Facebook groups (with participants enrolled in the first and second preclinical year, respectively) by combining the Facebook search function and word-of-mouth advice by enrolled students. The results of the additional interviews and focus groups confirmed that educational usage of Facebook among medical students in the first preclinical years almost exclusively takes place in these groups. Both closed groups used self-identifying names, stating the university (LMU Munich), study subject (medicine), and year of the cohorts’ initial semester (October 2012 or October 2013, respectively).

The groups were initiated by students and required an application for membership, followed by the acceptance through users already in the group. Students formed the first group we studied in their first preclinical semester, 2 or 3 weeks before the first official university event.

### Study Design

A multi-method approach was applied to answer the aforementioned research objectives. For characterization of medical students involved in Facebook groups, we conducted focus groups among Facebook users and structured interviews of specific student groups (*social media drivers* and students not using Facebook here called *new minorities*). For the evaluation of posting behavior and for identification of covered topics, we combined qualitative and quantitative methods to analyze posts in two semester-spanning Facebook groups. Data collection took place after completion of the winter semester in February 2014.

### Group Data Extraction

Groups were double checked by comparing the list of participants with the list of students enrolled in the respective preclinical semester.

All posts and comments of one academic semester (September 2013 to February 2014) were extracted using a custom script leveraging the Facebook Graph API for both groups, which were termed first preclinical year (PCY1) and second preclinical year (PCY2). Raw data contained content, poster identity document, and date for primary posts and replies. Further analysis was implemented in Excel 2010 (Microsoft).

### Qualitative and Quantitative Facebook Data Analysis

Due to expected saturation of data, we applied a thematic and content analysis of 10% of 1246 (PCY1) and 1168 (PCY2) total primary posts in each group over the course of one semester. The 10% analyzed primary posts were randomly chosen from all primary posts throughout the whole semester to avoid selection bias. Two experienced members of the research team independently defined categories with anchoring examples. Nine main categories were defined in the final general coding scheme.

**Figure 1:**
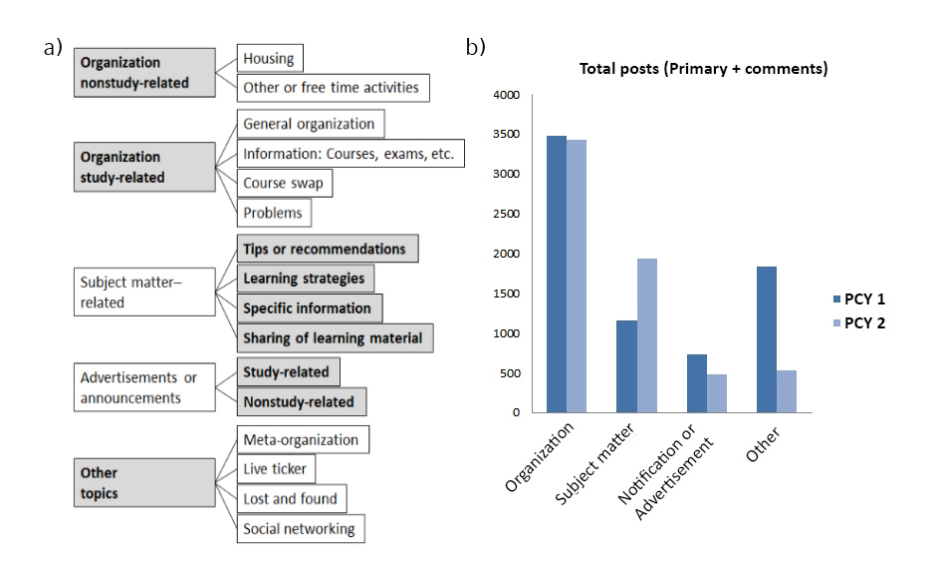
(a) Qualitative coding scheme for preclinical year 1 (PCY1) and preclinical year 2 (PCY2) groups. Categories and subordinate categories were identified. The shaded boxes indicate categories used for quantitative coding. (b) Number of total posts (primary posts and comments) in PCY1 and PCY2, grouped into the four dominant categories.

This scheme was used for coding the remaining 90% of posts and for quantification thereof (see Figure 1). Our approach provided insight into the absolute and relative (per week over the course of the semester) posting frequencies in the most abundant and relevant categories. If a post complex did not fit into the defined predominant categories, it was assigned to the category “other.” In a second coding step, all categories including the newly identified posts assigned to “other” were reevaluated. This ensured that no relevant topics were overlooked and guaranteed a thorough representation of the groups’ content. Postings were further categorized into “questions” and “statements.” For “questions,” we examined quantitatively the number of received responses and assessed qualitatively the relevance of the answers. Answers were categorized as “sufficient” and “not sufficient.” Questions were put in the category “sufficient” if the reply was constructive and relevant. Further quantitative analysis included the absolute number of posts, primary posts per week, average reply rates, and a classification of members according to their posting behavior (frequency).

Statistical Package for the Social Sciences (SPSS) version 23 (IBM Corp) was used for statistical analysis. Independent samples Mann Whitney *U* tests were used for posting frequency comparisons.

#### Focus Groups

To further assess motivation to join in Facebook groups, participation pattern, posting behavior, and efficiency, we conducted two semistructured focus groups using a precise focus group protocol to ensure consistency over various moderators and sessions (Multimedia Appendix 1) with medical students from our faculty (n=21). Students from preclinical year 1 and 2 were invited via email. Discussions were based on a protocol using open-ended questions on social media, Facebook and Facebook group usage, and user motivation (20 items).

#### Interviews

*Social media drivers* were defined as students with over 30 posts per semester, amounting to only 3.90% (62/1591) of total group members. The motivation and assessment of this subgroup was of particular interest to our research project. Hence, individuals identified by their extensive posting habits in the respective Facebook groups were invited to take part in semistructured interviews (n=4). For each subgroup, a detailed protocol was designed by two experienced authors (Multimedia Appendix 1).

Moreover, an outside perspective was gained by contacting students of medicine at the LMU Munich not registered on Facebook, so called *new minorities*. These students were identified through word-of-mouth and an email sent to the relevant semesters. Structured interviews focused on reasons for Facebook abstinence, and alternatives for information gathering were conducted (n=6).

All interviews and focus groups were audiorecorded and transcribed. For content analysis regarding the semistructured interviews and focus groups, two independent researchers developed a coding system by abstracting and categorizing the statements of the respective subgroups using MAXQDA (VERBI GmBH). Divergent coding was discussed and resolved.

The LMU ethics committee reviewed the research design and exempted the study from additional ethical approval. Confidentiality and anonymity with regard to electronic data was maintained throughout the study. Any names or potentially identifying information were removed before analyzing the data. The authors had no personal connections to the groups or were not registered members of the groups studied. In addition, authors involved in data analysis only had anonymized data to their disposal. Quotes were all translated from German to English for this manuscript. Pseudonyms were used to maintain confidentiality and anonymity.

## Results

### Demographics

At the time of data extraction, the PCY1 group counted 1213 members, with 71.2% (n=863) of members contributing at least one post per semester, named *active users*. The PCY2 group consisted of a total of 1149 members, with 63.36% (n=728) *active users.* The corresponding student cohorts enrolled in PCY1 and PCY2 at LMU Munich listed a total of 950 PCY1 students (58.9% [560/950] female) and a total of 966 PCY2 students (59.0% [570/966] female).

Approximately 6000 posts were extracted from each group, with about one-fifth of posts representing primary posts (see Table 1).

### User Typologies and Motivation

#### Focus Groups

All focus group participants (n=21, 12 female, and 9 male students) were registered on Facebook and were using semester-spanning groups. Motivation for setting up a Facebook account was mainly socializing and staying in touch with (international) friends and acquaintances. Nevertheless, 2 students registered solely for being able to join the aforementioned student groups. Students agreed that the overwhelming majority of their cohort were members in the respective groups. Medical students in their semesters that were not members of Facebook represented “[...] isolated cases,” as stated by a participant. When asked about relevant subgroups defined by posting behavior, one student stated that “[...] there is always the same 50 people that are very active [...].”

Students expressed that in semester groups, mainly student-related topics were covered and that organization-related posts were prominent among these. Apart from that, technical- and content-related information was regarded as important. Others recalled that content included scripts, exam questions, lecture slides, advertisements, course swaps, and selling of material such as medical books.

**Table 1:** Group demographics and characteristics. Demographics of the analyzed preclinical year 1 (PCY1) and preclinical year 2 (PCY2) group. Active users were defined as users with at least one post per semester. For user typology, user activity was divided into arbitrary groups, each contributing about one-third of posts. We defined social media drivers as members with >30 posts per semester, frequent posters with 11 to 30 posts, and lurkers with 1 to 10 posts per semester.

Group characteristics		Preclinical year 1	Preclinical year 2
Active users, n (%)		863 (71.15)	728 (63.40)
**User type distribution among active users, n (%)**			
	Social media drivers	28 (3.2)	34 (4.7)
	Frequent posters	130 (15.1)	131 (18.0)
	Lurkers	705 (81.7)	563 (77.3)
**Post contribution (of total posts), n (%)**			
	Social media drivers	1286 (21.70)	1718 (28.99)
	Frequent posters	2270 (38.31)	2300 (38.81)
	Lurkers	2370 (39.99)	1909 (32.21)
**Total posts**			
	Primary posts, n (%)	1168 (19.71)	1246 (21.02)
	Average comment or primary post	4.1	3.8

Students’ perspective on benefits and limitations of study-related Facebook use is detailed in Table 2. Interestingly, participation in Facebook groups was seen as efficient and time-consuming at the same time; efficient because of the ease of access and its usability, enabling group visits while spending leisure time on Facebook (students spent their time on Facebook anyway and could quickly check news and updates in the relevant groups). They felt it was time-consuming because to stay up-to-date, a significant amount of time is necessary to scan through the abundance of posts and comments. Implementation of a similar group on faculty websites was seen as problematic, as students said they would not post as freely and would “[...] feel supervised [...]”.

#### Social Media Drivers

In semistructured interviews (n=4), this subgroup reported using Facebook for educational as well as private purposes on a daily basis. When asked about their behavior and motivation for extensive posting in the respective groups, we identified two distinct subtypes:

Some *social media drivers* we interviewed see themselves as service providers, answering questions and providing information as well as resources. Altruism was named as the main reason for this dedication (“[...] it might help someone!”). Further reasons were access to exclusive information (“I am in a rather unique situation since I am not only a student but also work [at this institute] [...].”), as well as the feeling of an obligation to return a favor (“Because in the beginning, I benefited as well.”).

The second subtype comprised students that perform below average and use Facebook groups to get support. Contrary to the first subtype that contributes in sharing information, this subgroup seems to post more questions. For example, one student stated, “I was very [active], since I repeatedly had questions concerning upcoming exams.” The interviewed students did not express concerns regarding excessive peer pressure, ridicule, or unqualified answers to their numerous questions.

Social media drivers were aware of the imbalance of providing and consuming resources on Facebook and the consecutive varying roles of students in those groups. However, they did not see this nor “steering” the group in certain directions as problematic. From their perspective, the heterogeneity does not interfere with functionality. However, even this preselected group is experiencing limitations when it comes to organizing and administrating posts to maintain a structured way of presenting gathered information. The overall relevance of Facebook for academic outcome was evaluated to be significant (“I do think that access to important information is limited for people not participating in Facebook groups [...]”).

#### New Minorities

The interviewed students in that subgroup (n=6) were not members of Facebook at the time of the study, but most used other elements of social media such as Twitter and WhatsApp. They are aware of their minority status regarding (educational) Facebook usage and acknowledged Facebook groups as a highly relevant source for study-related content. One student even stated that “[...] I have to say I can’t directly compensate the losses [in study-related info or material]” he suffered by not participating in Facebook groups.

When asked for reasons for their behavior, they mainly expressed concerns also mentioned by Facebook users in focus groups (Table 2). Prominent reasons were privacy concerns, permanent availability, distraction from studying, and loss of valuable time.

**Table 2:** Qualitative content analysis—students’ perspective on benefits and limitations of study-related Facebook groups. Through a qualitative content analysis, benefits and limitations of Facebook groups were extracted from conducted interviews and focus groups.

Categories and subcategories			Anchoring example
**Limitations**			
	**Study-related**		
		Mutual dependence	*[...] You can’t rely on that. Simultaneously, one always has to look for answers elsewhere [...].*
		Information overload or disorganization	*When I am looking for a certain post—and I know it has to be somewhere, but I don’t know when it was posted—[...]. For me, that’s annoying.*
		Factual knowledge	*[...] once you take social media and Facebook [...] as your only source for medical education [...] a lot gets lost.*
		Peer pressure	*From my perspective, there is a lot of hysteria going on. Sometimes, not being confronted with that is not a bad thing.*
		Reliability	*Anyone can write something. In the end, you have to find a [reliable] source by your own.*
	**General**		
		Rudeness	*It escalated! People were rude and abusive to individuals that actually committed themselves to the group.*
		Commercialization	*In my eyes, it [marketing] has no place in groups like that.*
		Permanent availability	*[...] the aspect of permanent availability [...] are of high relevance why people say to prefer real social interactions instead of the Internet.*
		Distraction	*I have recognized that my concentration is severely compromised.*
		Anonymity	*It [Facebook group] is too big [...]. There is no feeling of togetherness.*
		Misleading presentations	*Everyone is presenting himself as one wishes to be, and not how one is in reality.*
		Mismatch with introverted personalities	*I am not the type of guy that uses social networks as they are designed.*
		Limited protection of privacy	*I do have some concerns about the privacy policy.*
		Dependence and irrational involvement	*The benefit of not being a user on Facebook is that I [...] don’t get lost and waste my time.*
**Benefits**			
	**Study-related**		
		Limited control by faculty or dark net	*Technically, one would not even be allowed to publish lecture notes online due to copyright reasons.*
		Collective knowledge	*Just think of it. One group has about a thousand members. Given that, there will always be one who knows the answer.*
		Established platform	*The vast majority of students are using Facebook. Thus there is no need to establish a new network.*
		Free of charge	*[...] information on Facebook is free of charge.*
		Effectiveness	*I save time since I get to information more quickly.*
		Connecting individuals with mutual interests	*One can easily organize groups when there are problems [...] since one can directly and quickly contact a lot of people [...].*
		Exclusive information	*I can tell from a friend, who is not using Facebook, that she has disadvantages because some resources can only be found there [Facebook] [...].*
	**General**		
		Mass media	*It is a good thing that everyone is on Facebook.*
		Intuitive handling	*I think it is a cool platform [...] to have an easily exchange with others.*
		Mobility	*[...] and it is a big advantage with smartphones these days.*
		Innovation	*There have been a lot of modifications since I last visited Facebook.*
		Live blog	*All results [of exams| are online instantly, on Facebook.*
		Online databank	*[...] anything that was posted is saved. One can have access to that anytime.*
		Diversity	*That’s the advantage of the Internet. One can read a lot of opinions and then decide which one might be the most plausible.*
		Social network	*Social interactions in a society are extremely important. That’s why everyone loves this [Facebook].*

To access information their peers got through Facebook groups, they utilized alternate ways of communication and contacted different people, for example, writing emails to peers or contacting them in person. They relied on official university platforms and forums, bought printed versions of lecture notes in copy shops, and selectively contacted experts such as peers and faculty members to get support. Two students contacted their fellow students to explicitly gain peer-mediated access to Facebook group content. One student even practiced periodic registration and deregistration on Facebook.

### Discussed Topics

An overview of topics discussed in both groups is presented in Figure 1 and Table 3. Categories were very homogenous in PCY1 and PCY2, as we could not find qualitative differences in posting themes.

#### Qualitative Description of Topics

##### Organizational Issues

We identified two subcategories of posts concerning organizational themes: study- and nonstudy-related. The part of the groups’ posts addressing nonstudy-related organizational issues covered mainly housing in Munich or insurance. In addition, information concerning student jobs and leisure activities were also posted.

The most abundant posts referred to study-related organizational issues. Four subcategories could be identified: (1) general organizational issues (eg, questions concerning course attendance regulations, procedures in case of illness, semester schedule, and directions to classes); (2) Information regarding courses, exams, and clerkships (eg, content-related information, duration, point in time, and prerequisites); (3) problems (difficulty with log-in on university online platforms or overlaps in course schedule); and (4) course swapping.

**Table 3:** Qualitative content analysis—discussed topics. Qualitative content analysis of 10% of posts was used to classify posts. The evolved coding scheme was then applied to the remaining 90% of posts and supplemented to accommodate all posts. Categories, subcategories, and an anchoring example are depicted here.

Categories and subcategories		Anchoring example
**Nonstudy-related organization**		
	Housing	*Hi hi! Has anyone a room available from Jan/Feb onwards? Or knows someone? [...]*
	Jobs or free time activities	*Hi everyone! I’m looking for a hands-on medicine related job that can be done in addition to the studies—does anybody know anything in this direction?*
**Study-related organization**		
	General organization	*Where do (most) lectures and courses take place?*
	Course or exam or clerkship specific information	*Hi, could someone post where the biology course tomorrow at 9 o’clock takes place?*
	Problems and issues	*I cannot login into my account on mecum-online.de. Does anybody experience similar problems?*
	Course swapping	*I need a partner to swap my biochemistry seminar at 12:30-14:00, I need the earlier one. Thanks*
**Subject matter**		
	Tips or recommendations	*Has anyone studied with the online Thieme learning program for the Biology exam and can tell me if it makes sense?*
	Learning strategies	*Is it true that if you buy the Prometheus [Anatomy] Atlas, you don’t need any other book?*
	Content specific info	*Hey people, has anyone discovered the solution for OIN Question 1?*
	Sharing of material	*Hi! Does anyone have the former Biochemistry II exam sheets at hand and could send me the solutions [...]*
**Notifications or advertisements**		
	Study related	*Come over to our MentoRing Fest! [...]*
	Nonstudy related	*You want a break of all the biochemistry stress and would like to party? Then you should join [Facebook event link]*
**Other**		
	Meta-organization	*[Facebook group link] for all students that are in Prof. Franks D1 [course] [...]*
	Live ticker	*Is someone right now in the reading hall and can tell me if there is space available? Somehow every [other] place is packed.*
	Lost and found	*Has someone found a white cotton cap with a white pompon at the uni? Maybe in the physiology EEG course room?! Is being heavily missed*!
	Social networking	*I’m also curious. Who of you is also a little bit older and what kind of schooling did you do [before you enrolled in medical school]?*

##### In Terms of Subject Matter

Most of these posts were related to the curriculum and therefore, also to the subjects being taught at the respective point in time. We further differentiated between tips or recommendations, learning strategies, contextual information, and material sharing.

Students referred to their cohort requesting individual tips, mostly on books and study material. In addition, questions concerning choice of electives were often posted. Others seek advice on learning strategies (which course to visit, when to start preparing for specific exams, and which study material to use). Apart from strategy and tips, content-related information on courses and exams was also shared and requested (questions left unanswered after the end of the lecture and unclear multiple choice questions).

Sharing of learning material and lecture notes could also be found in the Facebook groups we analyzed. This often consisted of exchange of exams or tests of previous years, including sample solutions, scripts, and even books.

##### Advertisements and Announcements

As shown in the focus group, advertisements and unsolicited notifications play a (detrimental) role in semester-spanning Facebook groups. We found a number of study-related advertisements that aimed at motivating students to participate in electives or other optional offers and commercial courses. Furthermore, group members looking for volunteers for research projects posted requests and compensation offers.

Nonstudy-related advertisements consisted of leisure activities and events, for example, announcements of parties, sale of concert tickets, and other extracurricular activities including nongovernmental organization call for action.

##### Other Topics

In several instances, members of the PCY1 group used this platform to post links to new Facebook groups of a course specific subgroup. In addition, students discussed about their communication through these groups, proposing rules or criticizing inappropriate behavior. This is an example of meta-organization. Another interesting finding was the use of the group as a *live blog*, asking for live updates on space availability in the medical library or progression of a lecture. In that sense, students also exchanged information about questions in oral exams, so that downstream examinees were better prepared. Furthermore, students used the group to assess their peers’ level of preparation for upcoming exams to compare it with their own level (this behavior was termed as peer-check).

We also found a use of the group as a *lost and found platform*, with students that found or lost personal items in campus associated buildings and areas posting notifications.

In addition, social networking was present in both groups and covered different aspects. For example, students looked through postings in the group for other students who shared certain characteristics. Some of the features mentioned were above average age, mother- or fatherhood, sports interest, music instrument, or common country or town of origin. In addition, the group was used to find and contact individuals that students had met in person.

The groups were used to increase economic efficiency. Students could easily identify others with whom they could share expensive medical books or car rides to similar destinations.

Moreover, the groups were used to voice and organize political interests of the cohort. In more than one instance, students mobilized using the groups to defend their interests toward faculty (in that particular semester eg, many posts referred to an ongoing conflict between students and the physics department).

Finally, an amount of posts with humorous content were posted. Students posted study-related images, interesting articles, or videos and jokes often related to extensive studying or clichés and stereotypes of the medical profession.

#### Quantification of Posting Patterns

**Figure 2:**
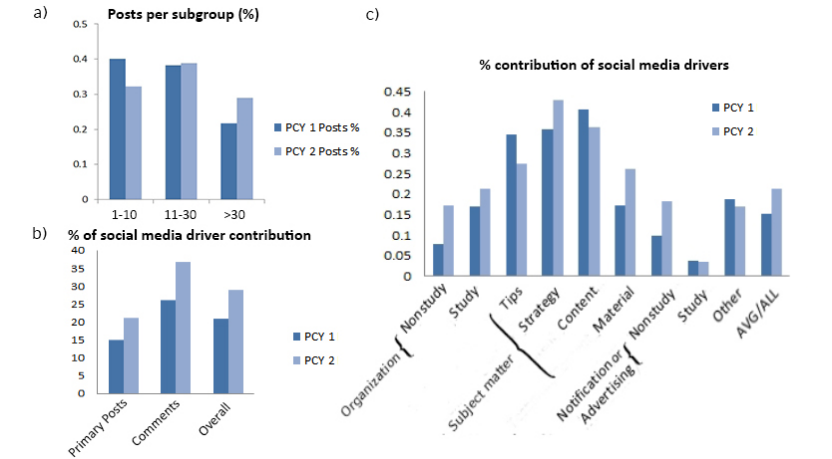
(a) Percentage of total posts in preclinical year 1 (PCY1) and preclinical year 2 (PCY2) contributed by subgroups that posted 1 to 10 (lurkers), 11 to 30 (frequent posters), and >30 posts (social media drivers) through the course of one semester. (b) Contribution of social media drivers (>30 posts) to primary posts, comments, and overall posts (primary and comments). (c) Contribution of social media drivers to identified categories.

Overall posts (primary posts and replies) amounted to 5926 in PCY1 and 5927 in PCY2, showing a sustained posting pattern in the second year (see Table 1). In PCY1 and PCY2, respectively, the large majority of active users contributed 1 to 10 posts, every sixth student contributed 11 to 30 posts, and only a small minority contributed over 30 posts (31-125 posts) through the course of the semester. Interestingly, members with 11 to 30 posts contributed almost the same amount of posts to PCY1 as to PCY2, whereas *social media drivers* (>30 posts) in PCY2 contributed notably more than in PCY1 (21.67%, 1284/5926 in PCY1 vs 29.00% 1719 of 5927 in PCY2 of all posts). Accordingly, students with limited amount of contributions posted less in PCY2 (40.04%, 2373/5926 in PCY1 and 32.17%, 1907/5927 in PCY2; Figure 2).

Posts about organizational issues dominated both groups and were equally represented in the first and second year group (58.77%, 3483/5926 in PCY1 and 57.92%, 3433/5927 in PCY2). In contrast, posts on subject matters were almost twice as frequent in the second year group (19.52%, 1157/5926 in PCY1 and 32.73%, 1940/5927 in PCY2) and reflected the second most represented category in that group. On the other hand, students in the first year group posted more about social networking, free-time activities, and sharing of nonstudy-related material (other topics: 31.10%, 1843/5926). Notifications or advertisements were represented relatively similar in both groups (12.50%, 741/5926 in PCY1 and 8.27%, 490/5927 in PCY2; Figure 1).

**Figure 3:**
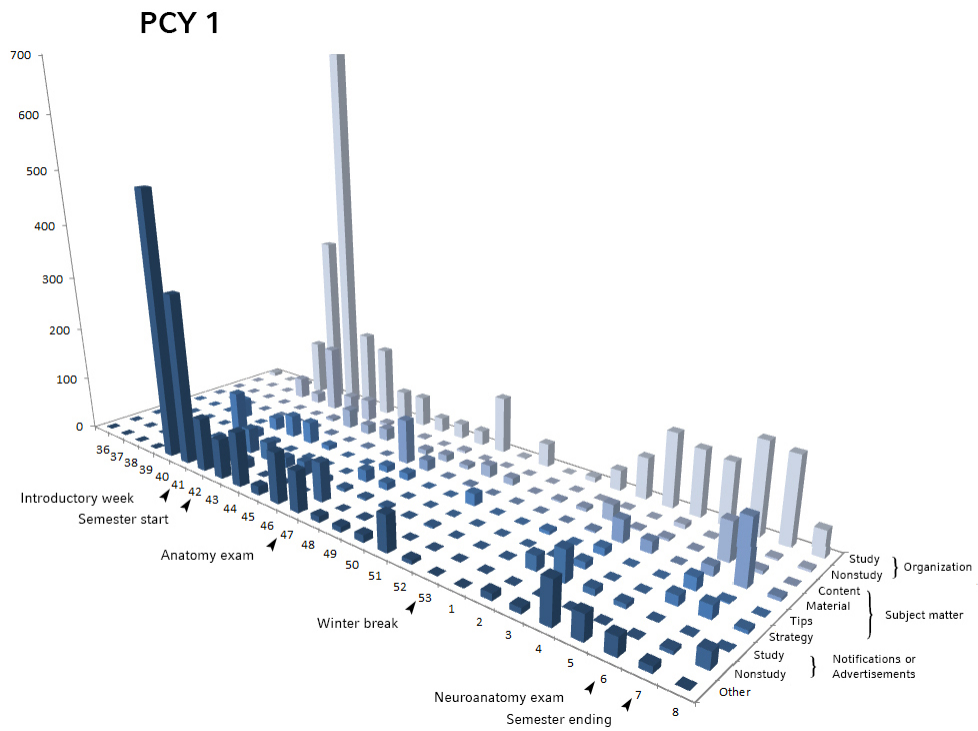
Number preclinical year 1 (PCY1) posts per semester week, divided into identified categories. Time axis (weeks) shows relevant semester events (arrows).

**Figure 4:**
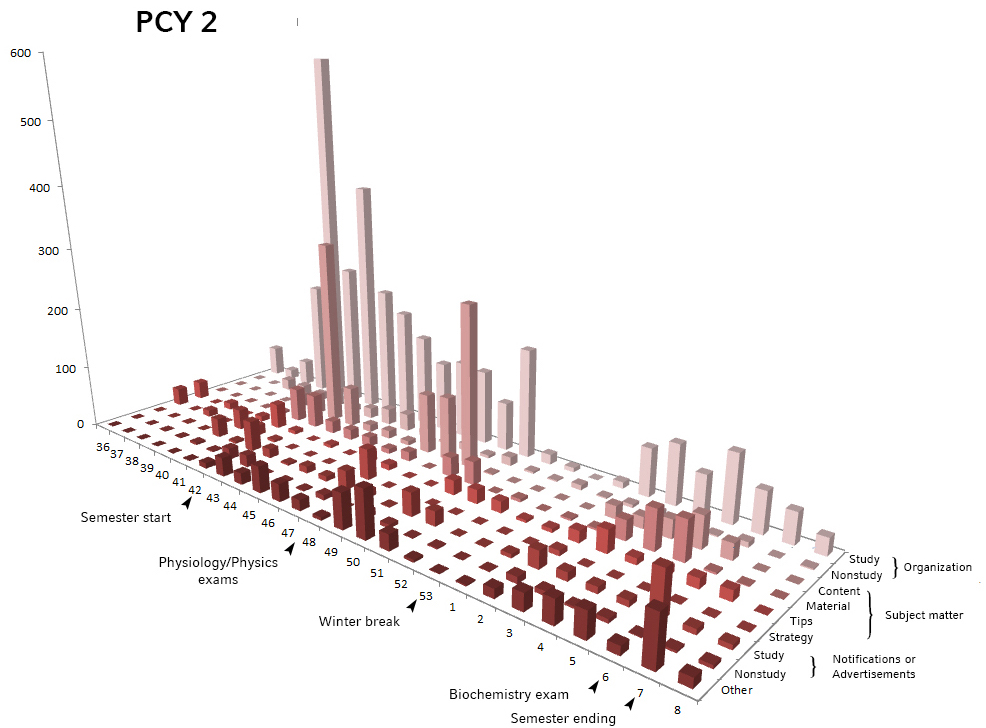
Number preclinical year 2 (PCY2) posts per semester week, divided into identified categories. Time axis (weeks) shows relevant semester events (arrows).

By correlating the posting frequencies in all nine main categories we defined over time with exams and other major semester milestones, we found a strong correlation between posting behavior and external events (Figures 3 and 4). As seen in Figures 3 and 4, in both groups, most organizational-related posts occurred within weeks of the beginning of the semester. The percentage of posts concerning organizational issues was higher in the first 3 weeks than in other weeks of the semester (PCY1: 245.2 posts vs 74.9 posts, *P*=.05 and PCY2: 298.0 posts vs 88.4 posts, *P*<.001).

Posts concerning subject matter peaked in weeks before the exams. In PCY1, we saw a maximum number of posts on the subject matter during weeks 47 and 7, correlating to the first exams of anatomy and neuroanatomy, respectively. In PCY2, posts on subject matter clustered in week 42, which corresponds to the biology exam, as well as the start of the physics practical course and in weeks 47/48, when PCY2 students took their physics and physiology exam (average 227.8 posts during these weeks vs semester average 34.5 posts, *P*=.001 on subject matter). To confirm our assumption, we qualitatively looked in to these weeks and verified that these topics are indeed predominant.

In both groups, frequencies in all categories dropped in calendar weeks 52, 53, and 1, which corresponds to the Christmas and New Year semester break (PCY1: semester average 217.6 posts vs semester-break average 23.3 posts, *P*=.002; PCY2: 294.2 posts vs 24.3 posts, *P*=.004).

**Figure 5:**
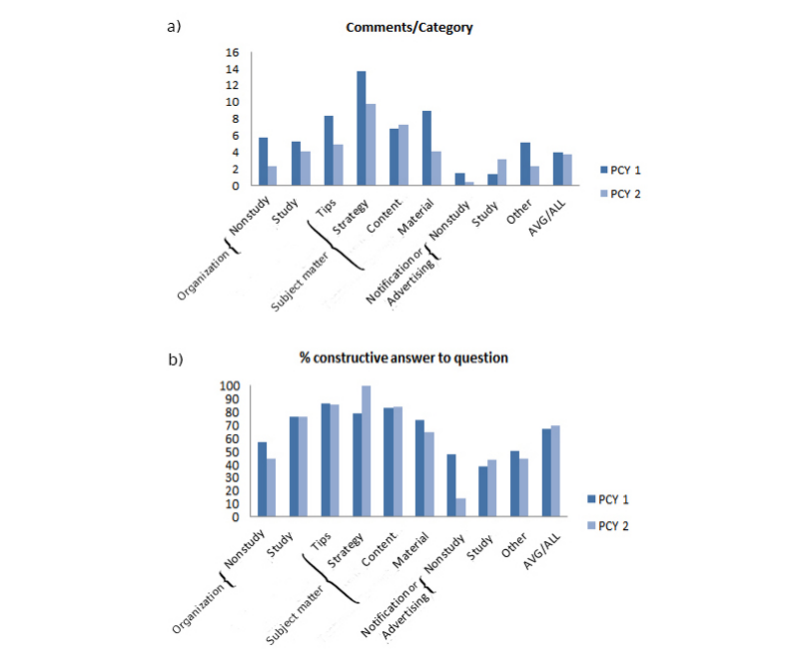
(a) In-detail analysis of comments per primary post, in all identified, coded categories. (b) Replies coded for percentage of constructive answers to questions in different categories.

We found that in the first year, 63.13% (738/1169), and in the second year, 77.13% (961/1246) of the total primary posts were questions. Overall, 67.2% (496/738) and 69.8% (671/961), respectively of these questions received satisfactory replies. Questions in the categories “notification” or “other topics” were answered in half or less of cases, whereas questions on subject matter were sufficiently answered in 78% to 100% of cases, depending on subcategory and semester. Study-related organizational questions, which were the bulk of all posts, were answered in 75.9% (341/449) and 76.2% (428/562) of cases in PCY1 and PCY2 groups (Figure 5).

A similar pattern was apparent when counting average replies per category (Figure 5). The overall average per primary post amounted to 4.1 replies in PCY1 and 3.8 replies in PCY2 and was heterogeneous among the subcategories. In PCY1, posts on subject matter received between 8.4 (tips) and 13.7 (strategy) comments, notifications or advertising 1.5, questions on organizational issues 5.3 to 5.8, and other topics 5.3 comments on average. In PCY2, posts on subject matter received between 4.1 (material) and 9.8 (strategy) comments, notifications or advertising 3.2, questions on organizational issues 2.3 (nonstudy-related) to 5.8 (study-related), and other topics 2.4 comments on average.

In both PCY1 and PCY2, *social media drivers* contributed more replies (22.59%, 1072/4757 and 31.08%, 1455/4681 respectively) than primary posts (15.06%, 176/1169 and 21.19%, 264/1246, respectively) in comparison to their 21.67% (1284/5926) and 29.00% (1719/5927) overall contribution (Figure 2). Analysis of subcategories revealed a similar pattern to the general post and reply average of *social media drivers*, with most involvement in posts on subject matter (strategy: 39%, 11/28; content: 37.3%, 63/169; and tips: 30%, 24/80) and lowest contribution to notification or advertising (7.5%, 35/465; Figure 2).

## Discussion

### Facebook Groups Play an Important Role in Students’ Lives

Facebook groups seem to have evolved as the main online social communities for medical students at LMU to complement the curriculum and to discuss study-related content. User typology revealed social media drivers (>30 posts per semester) as engines of group function, frequent users (11-30 posts), and a majority of average users acting rather as consumers or lurkers (1-10 posts). A wide range of topics is covered with a dominance of organization-related posts. By measuring reply rates and comments per category, we were able to highlight learning tips and strategies, material sharing, and course content discussions to be strengths of these groups. These Facebook groups are self-organizing and quickly adapt to organizational or subject-related challenges posed by the curriculum.

In line with a number of studies that show an increase in students’ usage of social media for educational purposes [15,16,19,33], our study confirms widespread use of Facebook at a large medical faculty not limited to this purpose. Although there are many ways to use social media (and Facebook in particular [8]), our results show that students in our faculty mainly use Facebook groups. This is supported by the fact that almost all students in one preclinical year are members of the respective semester-spanning group. The amount of questions and comments posted throughout the whole semester demonstrates continuous usage. Group members stating “without the (Facebook) group I wouldn’t survive medical school” or a nonmember commenting “[...] I have to say I can’t directly compensate the losses [in study-related info or material],” combined with the fact that some students only register on Facebook to participate in these groups, demonstrate the importance medical students ascribe to these groups. In a similar study performed at the Coalsville School of Social Sciences, students seem to predominantly use the wall function rather than Facebook groups. The difference could be because of the different structure of the curriculum or the time separation between the studies (2006 or 2007 vs 2013 or 2014) [9,11].

Although some of Mason’s essential attributes for educational usage of social media, such as communication, material sharing, and peer feedback can also be found in blogs, wall posts, and forums, these elements can be conveniently implemented using Facebook groups [12]. As seen in this study, the high frequency of questions and comments posted and the swiftness of replies show a considerable flow of information. Aggregation of information is obviously occurring given the amount of shared material, knowledge, and experience present. Finally, as each member of these groups had the means to read posts and react to them (complement, amend, or adjust), modification of informational content was also easily possible. Our work therefore supports Lee´s and McLoughlin’s assumption that Facebook groups could contribute a lot to creation of new content and could consequently play an important role as pedagogical tools [10].

Next to an educational role deriving from content-related posts, exchange of learning strategies, and feedback, an additional educational value concerning new media literacy skills can be postulated. According to Jenkins, the participation in online social media leads to creation and sharing of information, as well as collaboration with associated individuals. He defined these skills as vital for learning [34]. Students using the described Facebook-Groups presumably get to practice some of the literacy skills such as judgment, multitasking, collective intelligence, navigation, networking, and negotiation. This would be in line with Ahns findings, who examined (using learning analytics) how participatory behaviors correlate with acquisition of new media literacy skills [35]. Due to the lack of individual information (further SNS behavior) concerning group members, learning analytics could not be applied in our case. Further studies should look deeper into that aspect of Facebook-Group use.

### Four Main Types of Facebook Group Users Are Identified

Virtually all medical students in our faculty seem to be using Facebook groups, if only as passive consumers. It is remarkable that the majority of students in both groups contributed at least one post throughout the semester. The higher number of members in the groups compared with the respective, official student enrollment in each semester could be because of older students joining the group for support and information. For example, the student council encourages older students participating in a first year peer mentoring program to join the respective groups to share information and organize meetings. This might actually add a beneficial vertical axis in information sharing and support.

We identified four main types of students: (1) *new minorities* (students not participating in Facebook groups), (2) *Lurkers* (students joining the groups but hardly participating by posting), (3) *Frequent users* (students contributing above 10 posts throughout the semester), and (4) *Social media drivers* (students with an extensive contribution of more than 30 posts throughout the semester).

Interviews with *social media drivers* revealed a heterogeneous group of students who either posted because of an above average need for help and assistance or to contribute and help other students. These findings are similar to the findings of Kumar et al (2010), albeit in a different context [7].

An imbalance in contribution, as present in this case, could create an environment dominated by few, which dictate topics and discussions. However, we identified three arguments making a strong case against this assumption: (1) Posting analysis revealed that *social media drivers* are more likely to comment on an existing post than to place a primary post, (2) Topic-wise, the distribution of their comments follows the overall trend, and finally (3) the percentage of constructive answers by *social media drivers* was high. Consistent with this, students we interviewed didn’t feel inept or constrained in posting questions and making remarks. Hence, we believe that this particular subgroup makes a positive contribution to the efficacy of the whole group.

Interestingly, although Facebook networks are normally formed based on existing offline social networks or even offline latent ties [36-38], Facebook groups created for educational purposes seem to differ in that aspect. Students formed the first group we studied in their first preclinical semester, 2 or 3 weeks before the first official university event. As a result, participants of the group did not know each other before interacting via the semester-spanning group. It is conceivable that the large amount of communication following formation of the group is attributable to the fact that these groups are the only source of study-related information in the first weeks. One could consecutively hypothesize that after students have made some personal acquaintances and have formed real life peer groups, these alternative sources of information would render the respective Facebook groups less relevant. However, in our study we could not find a reduction in usage between first and second preclinical year (similar amount of questions and comments, similar amount of active users, and similar distribution of postings throughout the semester). Therefore, we infer that offline networks have little effect on the usefulness of educational Facebook groups. Further research and different methodology is necessary to examine the influence of offline networks in the evolution of online educational social media groups.

It needs to be mentioned that the sample size for focus groups (n=21) and interviews (n=10) was small considering the amount of students involved in the group (n=2362), and we can therefore not exclude additional user types to be present. Nevertheless, saturation analysis showed extensive redundancy between the two focus groups.

### A Wide Range of Topics is Covered, With a Dominance of Organization-Related Posts

Through analyzing all posts in PCY1 and PCY2 over the course of one semester, we were able to qualitatively identify relevant topics, as well as quantitatively assess frequencies and posting patterns. By using a thematic content analysis approach, combining it with a thorough semester-long evaluation, we could reduce biases and get a more holistic impression.

All five themes that emerged in Selwyn’s study (2009) through analysis of Facebook wall activity at Coalsville [9,11] could also be identified in the analysis of Facebook group posts at our faculty. This is surprising, as exchange of information in the Selwyn study happened between students that personally knew each other offline, whereas in our study students, at least initially, were not personally acquainted. As Selwyn’s study described mere qualitative differences, the predominance of themes could not be compared. Nonetheless, the high consistency of content despite two different curricula, subjects, and countries suggests that a global framework can be applied for students’ educational use of Facebook.

The majority of information exchanged in both groups we examined concerned organizational issues. The almost identical amount of posts in that category found in both years reveals a continuous need for clarification and information. Although faculty websites and brochures provide sufficient information for most of the issues raised, students preferred posting questions in Facebook groups. This is likely because of the immediacy, comprehensibility, as well as the accuracy of replies.

In contrast to the constant amount of posts concerning organizational issues, students in the second year group post a lot more about subject matter. We found that students discuss learning strategies or even explicit learning content. Most likely, this is because of the increasing conceptual and subject specific challenges over the course of preclinical medical studies and the upcoming first state exam after the 2nd preclinical year. Focus groups and interviews revealed the relevance of this aspect. Especially, sharing of learning materials such as scripts or exam questions was named as one of the main reasons for using these groups. Individuals even stated that Facebook was absolutely mandatory for their academic success in medical school. Additionally, the higher rate of overall replies and constructive answers to questions in this category (78% (11/14) to 100% (13/13)) underline the importance as well as strength of Facebook groups in this regard. These results highlight the role of social media in peer education and learning in accordance with findings in the literature [15,21,25,39-42].

In addition to the categories we analyzed, the groups are also used for a broad range of nonstudy-related content. For example, we identified a number of peer-mentoring elements as an interface between education and extracurricular aspects in a preceding study, that is, providing emotional support: “It’s absolutely normal to be afraid of the terminology exam and the Latin grammar questions, but it is really easier than you think” [19]. Considering the amount and diversity of issues raised, we conclude that these groups serve as a broad platform for a variety of content.

### Semester-Spanning Facebook Groups Are Highly Dynamic and Show Plasticity

Our in-depth quantitative analysis of posting patterns reveals specific posting patterns over each semester.

The use of Facebook as a *live blog*, for example, concerning currently free seats in the library or questions asked in an oral exam minutes away, exemplifies the dynamics and quick response rates associated with the use of Facebook groups. Not only posts and comments but also posting patterns were highly dynamic. We could show that overall post frequencies and covered topics adapt to current events. As probably most students in each cohort will face comparable challenges and have similar questions at the same time period, this plasticity helps the group to be relevant at any given point in time.

Furthermore, by sharing information so easily, it seems sufficient if only a few members of the group spent time checking primary sources. In effect, this construct contributes to the efficiency of the whole group, as already described for social media in companies [43].

Tuckman’s model of group development consists of four phases: forming, storming, norming, and performing [44]. Essentially, one would expect that newly formed Facebook groups would undergo consolidation over time and therefore, function better at later points in time. In the light of this theoretical framework, the increase in members contributing over 30 posts along with a decrease in students contributing 1 to 10 posts in the second year could be consistent with group evolution. Additionally, the changes in predominant schemes (content- and subject-related posts increased, whereas total posts classified in “other” dropped to roughly one-third, accompanied by a drop in notifications or advertisements) could also hint at a more streamlined discussion and flow of information. Along the same lines, primary posts that include questions increased, as well as the contribution of *social media drivers* to primary posts and replies. We conclude that these groups seem to undergo considerable development over time, which can be explained with phases of group formation according to group theory [44,45].

### Multiple Limitations of the Described Use of Facebook Are Identified

In accordance with previous studies, we identified potential limitations of Facebook groups for educational use. First, privacy issues were prominently expressed by all students we interviewed, especially by the cohort of *new minorities,* and were frequently named as a reason against joining Facebook (and the groups). Students mainly felt their personal data were at risk. Professionalism issues seem to be more relevant in groups formed by students in the clinical parts of their studies, as information could involve patient data [23,46]. This could have not only personal but also legal implications, clearly limiting the use of these groups in contrast to university-hosted, protected platforms, which are already in use at some institutions [14,47].

Furthermore, the identification of even a small percentage of students not enrolled in Facebook for understandable reasons prohibits the option of using these groups as an official platform for the respective semesters.

Moreover, Facebook groups are clearly not designed and programmed for educational purposes. Students complained about technical limitations, which make it hard to organize, share, and find information. For example, similar questions were often posted multiple times. This limits the use of the group as a database. In line with Madge et al (2009), students also described the platform itself as distracting and time-consuming by mixing study-related and private content [48].

Finally, Facebook groups can be easily misused for commercial interests [33,49]. We identified a substantial amount of advertisements in both groups. In a time of personalized advertisements, a homogenous group of students is a very valuable target and could not only lead to distracting advertisement but also hidden product placement, for example, recommendations for certain books or other learning materials.

### Conclusions

We found semester-spanning Facebook groups to be an essential part of the learning environment for most medical students at LMU Munich. A wide range of study-related topics were covered; organizational posts and posts with regard to subject matter seemed predominant. The reach, involvement of students, plasticity, and dynamics make these groups very powerful knowledge bases, as well as platforms for posing questions and starting discussions on a wide range of topics.

Faculty could cover many aspects that are discussed in students’ Facebook groups, especially the bulk of organizational questions and posts. However, the dynamics, plasticity, and response time of social media is difficult to match. Nevertheless, faculty could benefit from these groups and use them to their advantage. For example, universities could feed relevant information to the groups, increasing their reach and interacting more closely and directly with their students. Moreover, posting patterns concerning certain topics could be used to identify common problems and understanding semester-related dynamics, reacting more quickly and precisely. Information could be structured better, and organizational deficits could be easily identified. By reviewing discussed topics, the curriculum could be adapted to challenging teaching and learning content that posed problems to students. The peer teaching aspect could be greatly enhanced by introducing trained senior students providing help and assistance to their younger peers. This could enhance vertical knowledge transfer and information quality at the same time. Moreover, in a less anonymized setting, student with weaker performance could be identified earlier and be supported adequately by mentors and tutors. Curriculum structure, organization, and content are subjects to instantaneous feedback in these groups, allowing for quick adaptations and possibly replacing costly evaluation forms and surveys. Nevertheless, more research is necessary to assess the influence of possible participation of faculty members in these groups, as social media has also been identified as an opportunity to vent study-related frustrations [31].

Additionally, other issues such as the quality of posts, privacy, and knowledge conservation should be addressed before faculties could get more actively involved in Facebook groups [23].

## Appendix

Multimedia Appendix 1 Interview guidelines.

## Abbreviations

LMU: Ludwig-Maximilians-University
PCY: preclinical year
SNS: social networking site
