# Supplementary material for: Facebook Groups as a Powerful and Dynamic Tool in Medical Education: Mixed-Method Study
Source: J Med Internet Res. 2017 Dec 22;19(12):e408. doi: 10.2196/jmir.7990 (PMC5756318; doi:10.2196/jmir.7990)
Supplement: Multimedia Appendix 1 [file jmir_v19i12e408_app1.pdf]

Guideline for interviews with New Minorities

Introduction

Introduction of the interviewer

Purpose of the study and use of data

Ground rules

Estimated length of the interview (20-30 min)

Consent and options to withdraw

Confidentiality

Topic 1: Confirming new minority status

What year of medical school are you in and how old are you?

Which social networks are you a member of?

PROBE: Are you a member of Facebook?

PROBE: Do you use WhatsApp or Twitter?

PROBE: Have you ever been a member of social networks other than the ones previously mentioned?

Topic 2: Facebook in medical education

What is your opinion on social media in general?

PROBE: Do you think it is useful to stay in touch with friends worldwide?

PROBE: Are you concerned about privacy issues?

More particularly, what is your opinion on Facebook?

PROBE: What are the reasons for the fact that you are not a member of Facebook?

PROBE: Do you have concerns over privacy issues?

PROBE: Do you feel like you could be wasting your time spending time on Facebook?

PROBE: Why do you think other people, and medical students in particular, are not on Facebook? Can you imagine possible reasons prompting people to delete their account?

Since you are not a member of Facebook, are you aware of its structure and mechanisms?

PROBE: Do you know how the user interface looks like?

In general, what role do you think social media play in the context of medical education?

PROBE: Do you think it plays a vital role in our curriculum at LMU?

More particularly, what role do you think Facebook plays in the context of medical education?

PROBE: From your perspective, what benefits are your fellow students hoping to get by being active members on Facebook?

How do you compensate for the loss of the benefits you mentioned?

PROBE: What kind of resources do you normally use?

PROBE: Would you have concerns over the validity of the information being posted in closed groups on Facebook?

PROBE: According to your estimate, how many of your fellow students are members of Facebook?

In summary, what do you think are the benefits and limitations of choosing not to be a member of Facebook?

Ending questions

Do you want to add anything regarding this topic?

Do you feel like we missed important issues with regards to social media in medical education?

How do you feel about the interview? Would you like to give us feedback?

Guideline for interviews with Social Media Drivers

Introduction

Introduction of the interviewer

Purpose of the study and use of data

Ground rules

Estimated length of the interview (20-30min)

Consent and options to withdraw

Confidentiality

Topic 1: Use of social media and social habits

What year of medical school are you in and how old are you?

Which social networks are you a member of?

PROBE: Are you a member of Facebook?

PROBE: Do you use WhatsApp or Twitter?

PROBE: Have you ever been a member of social networks other than the ones previously mentioned?

Do you consider yourself to be a social person with a big circle of friends and acquaintances?

PROBE: How important is social support to you?

How do you see yourself with regards to academic performance?

PROBE: Do you think you are below or above average?

Topic 2: User typologies

Do you feel like there are different user typologies? Do you feel like people engage differently?

PROBE: Would you say that there are individuals who primarily consume information and are hardly active themselves?

PROBE: In this context, what role would you attribute to yourself?

What are the reasons for the fact that you are frequently posting study related content?

PROBE: Do you have benefits by doing so?

With members of the group contributing heterogeneously, do you see a problem with that?

PROBE: Do you think it is unfair that some individuals primarily consume without contributing??

Regarding your posts, do you feel responsible for the content?

PROBE: Do you double-check the validity of the information you are spreading?

What do you think about the arrangement of posts and related information in those groups?

PROBE: From your perspective, do you think the group would benefit from administrators?

PROBE: Do you feel like you are looking after the group as a moderator/administrator?

Topic 3: Facebook in medical education

What is your opinion on social media in general?

PROBE: Do you think it is useful to stay in touch with friends worldwide?

PROBE: Are you concerned about privacy issues?

More particularly, what is your opinion on Facebook?

PROBE: Do you have concerns over privacy issues?

PROBE: Do you feel like you could be wasting your time spending time on Facebook?

PROBE: Why do you think other people, and medical students in particular, are not on Facebook? Can you imagine possible reasons prompting people to delete their account?

In general, what role do you think social media play in the context of medical education?

PROBE: Do you think it plays a vital role in our curriculum at LMU?

More particularly, what role do you think Facebook plays in the context of medical education?

PROBE: From your perspective, what benefits are your fellow students hoping to get by being active members on Facebook?

PROBE: Would you have concerns over the validity of the information being posted in closed groups on Facebook?

PROBE: According to your estimate, how many of your fellow students are members of Facebook?

In summary, what do you think are the benefits and limitations of choosing not to be a member of Facebook?

Ending questions

Do you want to add anything regarding this topic?

Do you feel like we missed important issues with regards to social media in medical education?

How do you feel about the interview? Would you like to give us feedback?

Guideline for focus groups

Introduction hosts

Introduction of the interviewer

Purpose of the study and use of data

Ground rules

Estimated length of the interview (1h)

Consent and options to withdraw

Confidentiality

Introduction respondents

Can everyone please state his/her name and current year of medical school?

Which social networks are you a member of?

Does everyone of you have a Facebook account?

Transition questions

What are your personal reasons for being a member of Facebook?

What groups on Facebook are you a member of?
What study related groups are there and are you a member of those as well?
How many of your fellow students are members of such study-related groups?
What benefits do you expect to have by being members of those very groups?
Do you look for particular pieces of information when needed or do you rather observe developments on a regular basis?

Key questions

What happens in those groups?
- PROBE: What are the (main) topics being covered by the discussions?
- PROBE: Are there more study or non-study related posts?

How do people react to questions? How do people react to requests for support?
- PROBE: Is bullying an issue?
- PROBE: How long does it usually take until a question is answered?

How much do you contribute yourself to those groups? How does your support for other members look like?
- PROBE: What kind of resources do you share? Scripts, ebooks?

What kind of problems can be solved by posting a question to such groups? Are there any limitations?
- PROBE: Is the use of Facebook sufficient to solve most (study-related) problems?
- PROBE: What are alternative resources you would like to have?
- PROBE: Is there a true sense of emotional/social support or is it more limited to solving study-related problems?

What benefits/limitations can you think of with regards to Facebook and medical education?
- PROBE: Do the groups appear to be unorganized?
- PROBE: Do you have concerns about patient-related data and privacy?

Ending questions

What would you like to change with regards to the study related groups?
In this context, would you like to see more mentoring and support by the Medical Faculty?
